# Supplementary material for: High vaccination coverage and inadequate knowledge: Findings from a community-based cross-sectional study on Japanese Encephalitis in Yangon, Myanmar
Source: F1000Res. 2020 Sep 8;9:6. Originally published 2020 Jan 6. [Version 3] doi: 10.12688/f1000research.21702.3 (PMC7525338; doi:10.12688/f1000research.21702.3)
Supplement: Peer review history

# Peer review: Reviewer response for version 3

**DOI:** 10.5256/f1000research.29368.r71016

**Published:** 2020-09-29

**Authors:** Babasaheb V. Tandale, Pravin Deshmukh

**Affiliations:** 1 ICMR - National Institute of Virology, Pune, India; 2 ICMR-National Institute of Virology, Pune, Maharashtra, India

The review comments for version 2 have been clarified and responded. The manuscript revised version addresses the concerns raised earlier. The readers would have opportunity to understand and conclude accordingly.

Is the work clearly and accurately presented and does it cite the current literature?

Yes

If applicable, is the statistical analysis and its interpretation appropriate?

Partly

Are all the source data underlying the results available to ensure full reproducibility?

Partly

Is the study design appropriate and is the work technically sound?

Yes

Are the conclusions drawn adequately supported by the results?

Yes

Are sufficient details of methods and analysis provided to allow replication by others?

Yes

Reviewer Expertise:

Epidemiology of virus diseases causing outbreaks.

We confirm that we have read this submission and believe that we have an appropriate level of expertise to confirm that it is of an acceptable scientific standard.

# Peer review: Reviewer response for version 2

**DOI:** 10.5256/f1000research.27677.r66013

**Published:** 2020-08-03

**Authors:** Babasaheb V. Tandale, Pravin Deshmukh

**Affiliations:** 1 ICMR - National Institute of Virology, Pune, India; 2 ICMR-National Institute of Virology, Pune, Maharashtra, India

Comments on the revised manuscript:

1. As this study was aimed at estimation of awareness level and vaccination coverage level, the power of the study may not be adequate to consider the additional analyses using multiple logistic regressions and reporting of odds ratios. Therefore, authors may consider the power calculation retrospectively and report the same if appropriate based on power calculation.
2. The revised version also retained aim to describe awareness and coverage only. Authors may either change aim if the study has enough power to undertake associations and comparisons for identifying associated variables and risk factors by using multivariate analysis. Otherwise, the analytical component may be dropped from the results, if not appropriate and not enough power.
3. In Table 3 and 4, it would be important to provide the actual numbers along with percentages so as to verify the findings by the readers.
4. First chi square (χ2) association may be reported for table 3 and 4, if significant, then odds ratios may be indicated if the study power is adequate for the same.
5. High and low education has neither been defined in methods nor in foot note in table 3. It is also not clearly categorised in supplemental file associated with the manuscript.

Is the work clearly and accurately presented and does it cite the current literature?

Yes

If applicable, is the statistical analysis and its interpretation appropriate?

Partly

Are all the source data underlying the results available to ensure full reproducibility?

Partly

Is the study design appropriate and is the work technically sound?

Yes

Are the conclusions drawn adequately supported by the results?

Yes

Are sufficient details of methods and analysis provided to allow replication by others?

Yes

Reviewer Expertise:

Epidemiology of virus diseases causing outbreaks.

We confirm that we have read this submission and believe that we have an appropriate level of expertise to confirm that it is of an acceptable scientific standard, however we have significant reservations, as outlined above.

## References

[1] : High vaccination coverage, inadequate knowledge and high vector density: Findings from a community-based cross-sectional study on Japanese Encephalitis in Yangon, Myanmar. *F1000Research*.2020;9: 10.12688/f1000research.21702.2 10.12688/f1000research.21702.2 PMC752533933014343

# Response

**Published:** 2020-09-03

**Authors:** Pyae Phyo Kyaw

**Affiliations:** Department of Medical Research, Myanmar

Comment 1. As this study was aimed at estimation of awareness level and vaccination coverage level, the power of the study may not be adequate to consider the additional analyses using multiple logistic regressions and reporting of odds ratios. Therefore, authors may consider the power calculation retrospectively and report the same if appropriate based on power calculation.

 Response: Thank you for this comment. In the initial draft that was submitted, we did not perform regression analysis. We conducted an association between socio-demographic characteristics of parents/guardians with their knowledge level, and an association between knowledge level of parents/guardians with vaccination status of the children as a secondary analysis after a suggestion by one of the reviewers. We did not conduct post hoc or retrospective power analysis because power calculation after the data collection and effect size being observed do not usually provide true power for detecting statistical significance, and confidence intervals are more informative retrospective power calculations. Retrospective power calculation may inform for sample size calculation of future study but has little value for the current study. In addition, the practical significance by presenting effect size and 95% confidence interval are more informative than emphasizing on p-value and statistically significant. Hope you can agree with our stance on this.

 Comment 2. The revised version also retained aim to describe awareness and coverage only. Authors may either change aim if the study has enough power to undertake associations and comparisons for identifying associated variables and risk factors by using multivariate analysis. Otherwise, the analytical component may be dropped from the results, if not appropriate and not enough power.

 Response: Thanks for your comment. The primary aim of the study is still to describe the awareness and vaccination coverage as there is no study report this in region. We also decided to keep the regression analysis as a secondary analysis. I added objective three. (line no: 44-47)

 Comment 3. In Table 3 and 4, it would be important to provide the actual numbers along with percentages so as to verify the findings by the readers.

 Response: Thank you for this comment. As the OR and adjusted OR are outputs of weighted analysis, if we provide the number percentage (unweighted), it may not match with the weighted OR and adjusted OR. Hence we have avoided this. We hope this is fine. It is impossible to provide actual numbers along with percentages for the logistic analysis.

 Comment 4. First chi square (χ2) association may be reported for table 3 and 4, if significant, then odds ratios may be indicated if the study power is adequate for the same.

 Response: Thanks for this comment. We estimated odds ratios using logistic regression to estimate the association between parent characteristics and good knowledge. We did not perform chi-square test to avoid multiple testing issue. The crude / unadjusted logistic regression p value is similar to the chi-square p value. If crude / unadjusted p value <0.2, the variable was included in the logistic regression model. This way our model is parsimonious.

 Comment 5. High and low education has neither been defined in methods nor in foot note in table 3. It is also not clearly categorized in supplemental file associated with the manuscript.

 Response: Thanks for your comment about education level. High level education contains graduate and high school. Low level education contains middle school, primary school, can read and write and illiterate. It is stated in foot note of table 3. (line no:227-228)

# Peer review: Reviewer response for version 2

**DOI:** 10.5256/f1000research.27677.r66014

**Published:** 2020-07-01

**Authors:** Jing An

**Affiliations:** 1 Department of Microbiology, School of Basic Medical Sciences, Capital Medical University, Beijing, China

The revisions are accepted. I have no further comments to make.

Is the work clearly and accurately presented and does it cite the current literature?

Yes

If applicable, is the statistical analysis and its interpretation appropriate?

Partly

Are all the source data underlying the results available to ensure full reproducibility?

Yes

Is the study design appropriate and is the work technically sound?

Partly

Are the conclusions drawn adequately supported by the results?

Partly

Are sufficient details of methods and analysis provided to allow replication by others?

Yes

Reviewer Expertise:

Interaction between viruses and hosts; Mosquito-borne flavivirus; Vaccine

I confirm that I have read this submission and believe that I have an appropriate level of expertise to confirm that it is of an acceptable scientific standard.

# Peer review: Reviewer response for version 1

**DOI:** 10.5256/f1000research.23924.r63433

**Published:** 2020-05-29

**Authors:** Babasaheb V. Tandale, Pravin Deshmukh

**Affiliations:** 1 ICMR-National Institute of Virology, Pune, Maharashtra, India; 2 ICMR - National Institute of Virology, Pune, India

- The manuscript is composed well and has been presented in a very simple way making it very easy to understand by the readers. As indicated by the other reviewer, more in-depth analysis and comparison to explain the similarities and differences in knowledge and perception of JE disease and JE vaccine may be considered along with the explanatory variables for coverage of JE vaccination. The comparisons would thus help identify the important aspects that could be targeted for improvements. 
- The sampling design could be better explained with sampling ratios planned at different phases of sampling. 
- Vector distribution / density has been indicated in title, however it is not dealt in abstract, objectives, methods and results. Therefore, this aspect may be dropped from title also. It could be mentioned in discussion section as the secondary data and indicated for its importance for understanding of study findings.
- The higher coverage of vaccination in spite of low level of knowledge and perception of JE disease and JE vaccine is difficult to understand. It may be highlighted and discussed in details. 
- The objectives may be clearly mentioned at the end of background section. 
- The potential sources of bias in selection, verification of information and interpretation of information given by respondents needs to be critically appraised and discussed. The confounders also need to be addressed properly. 
- The verification of vaccination card for ascertaining vaccination recall needs to be addressed with efforts made for data quality. 
- The sample size and statistical analysis plan could be included with specific details on the basis of assumptions and proposed analysis. The sample size estimation could have been based on reported coverage of vaccination to be 93% in small survey findings earlier. The weighting approach needs to be provided clearly with its application in crude/unadjusted and adjusted/corrected coverage. 
- The survey currently presented is mostly descriptive in nature and needs to be made analytical with comparisons that could be made and presented. 
- The questionnaire validity may be presented with variable responses for similar aspects. 
- Non-responses and its handling in analysis may be included. 
- The baseline characteristics of respondents may be compared with good or bad knowledge, acceptable perception or otherwise and vaccinated and unvaccinated. 
- Limitations of study may be discussed with interpretations based on the same. 
- Generalisability of study findings may be considered and discussed. 

Is the work clearly and accurately presented and does it cite the current literature?

Yes

If applicable, is the statistical analysis and its interpretation appropriate?

Partly

Are all the source data underlying the results available to ensure full reproducibility?

Partly

Is the study design appropriate and is the work technically sound?

Yes

Are the conclusions drawn adequately supported by the results?

Yes

Are sufficient details of methods and analysis provided to allow replication by others?

Yes

Reviewer Expertise:

Epidemiology of virus diseases causing outbreaks.

We confirm that we have read this submission and believe that we have an appropriate level of expertise to confirm that it is of an acceptable scientific standard, however we have significant reservations, as outlined above.

# Response

**Published:** 2020-06-25

**Authors:** Pyae Phyo Kyaw

**Affiliations:** Department of Medical Research, Myanmar

1. The manuscript is composed well and has been presented in a very simple way making it very easy to understand by the readers. As indicated by the other reviewer, more in-depth analysis and comparison to explain the similarities and differences in knowledge and perception of JE disease and JE vaccine may be considered along with the explanatory variables for coverage of JE vaccination. The comparisons would thus help identify the important aspects that could be targeted for improvements. 

 Response: Thank you for your suggestion and we performed more in-depth analysis to determine the socio-demographic characteristics associated with good knowledge score and presented in the table-3. (line 218-234) We also performed an in-depth analysis to determine the good knowledge score with the child being vaccinated and presented in the table-4. (line 239-244)

 2. The sampling design could be better explained with sampling ratios planned at different phases of sampling. 

 Response: We added a detailed explanation of the sampling design and procedure in the method section. (line 81-105)

 3. Vector distribution / density has been indicated in title, however it is not dealt in abstract, objectives, methods and results. Therefore, this aspect may be dropped from title also. It could be mentioned in discussion section as the secondary data and indicated for its importance for understanding of study findings.

 Response: We have revised the title according to your suggestion.

 4. The higher coverage of vaccination in spite of low level of knowledge and perception of JE disease and JE vaccine is difficult to understand. It may be highlighted and discussed in details. 

 Response: We have added a discussion in the revised manuscript. (line 276-282)

 5. The objectives may be clearly mentioned at the end of background section. 

 Response: We revised the paragraph at the end of introduction section and stated the objectives clearly. (line 42-44)

 6. The potential sources of bias in selection, verification of information and interpretation of information given by respondents needs to be critically appraised and discussed. The confounders also need to be addressed properly. 

 Response: We have discussed the potential for recall bias as half the respondents could not produce the immunization or JE campaign card (for details see line 251-258). We performed more in-depth analysis to assess factors associated. (line 145-148) (line 218-226)

 7. The verification of vaccination card for ascertaining vaccination recall needs to be addressed with efforts made for data quality. 

 Response: We were able to verify 50% of the vaccinated children with their vaccination card. We acknowledged that the respondents who did not have vaccination card would subject to recall bias. (line 254-255)

 8. The sample size and statistical analysis plan could be included with specific details on the basis of assumptions and proposed analysis. The sample size estimation could have been based on reported coverage of vaccination to be 93% in small survey findings earlier. The weighting approach needs to be provided clearly with its application in crude/unadjusted and adjusted/corrected coverage. 

 Response: We agree that we should have calculated the sample size based on a previous estimate. But we decided to assume a coverage of 50% that would give a maximum sample size. We have added a detailed description on sample size calculation and statistical analysis in the revised manuscript. We provided weighted estimates as the analyses were weighted (inverse probability sampling) for the multi-stage sampling design, this was done both for the descriptive and analytical calculations (line 82-89, 136-137)

 9. The survey currently presented is mostly descriptive in nature and needs to be made analytical with comparisons that could be made and presented. 

 Response: We performed more in-depth analysis to determine the socio-demographic characteristics associated with good knowledge score and presented in the table-3.

 (line 218-240) We also performed an in-depth analysis to determine the good knowledge score with the child being vaccinated and presented in the table-4. (line 239-244)

 10. The questionnaire validity may be presented with variable responses for similar aspects. 

 Response: Thank you for the comment. Unfortunately, we do not have information on questionnaire validity.

 11. Non-responses and its handling in analysis may be included. 

 Response: There was no non-responses in our study because we went to the household using the sampling interval until the required sample size was reached. We added this information in the revised manuscript. (line 104-105)

 12. The baseline characteristics of respondents may be compared with good or bad knowledge, acceptable perception or otherwise and vaccinated and unvaccinated. 

 Response: We have added results on the association between the baseline characteristics of respondents with knowledge level and vaccination status. (line 218-240)

 13. Limitations of study may be discussed with interpretations based on the same. 

 Response: We have added this discussion in our revised manuscript. (line 250-258)

 14. Generalisability of study findings may be considered and discussed.

 Response: We have added this discussion in our revised manuscript. (line 250-258)

# Peer review: Reviewer response for version 1

**DOI:** 10.5256/f1000research.23924.r62066

**Published:** 2020-04-27

**Authors:** Jing An

**Affiliations:** 1 Department of Microbiology, School of Basic Medical Sciences, Capital Medical University, Beijing, China

The authors here conducted a set of survey data to reflect the public awareness on JEV in community. A great deal of data collection was presented. These contribute to show the significant progresses of the JE campaign in Yangon. However, there are several questions to be answered.

 Some Comments: 

1. The whole article was simple as a scientific research. More in-depth analysis of data is recommended. More comparison should be conducted to reflect the details in multi-aspects. For example, "parents/guardians had poor knowledge of JE" is sentenced. Since the details of the guardians were investigated, the correlation should be conducted, like with age? or occupations? or else? or none. Besides, are there any differences in awareness between urban and rural places?...
2. To fully reflect the effect of public awareness in Yangon, authors should not choose nine townships randomly. Especially the locations are concentrated. The choices need to be more meaningful, like the widest vector distribution ones, the most severely suffered ones. OR, at least, the details of these nine townships should be presented, like incidence cases.
3. The complex relationship between JEV and congeneric members, like DENV and ZIKV, is widely proposed. Since dengue is prevalent locally, the vaccination against Flavivirus members or infections should be also considered in the questionnaire.
4. In table 1, (1) is single parent family excluded? (2) what does "Do not know family members suffered JE" exactly mean? do not know the symptoms or do not know the children's conditions?
5. "23% did not know the symptoms of JE" is sentenced. So, how will these parents know their kids are not suffered from JE? Is the "0" morbidity presented by parents credible?

Is the work clearly and accurately presented and does it cite the current literature?

Yes

If applicable, is the statistical analysis and its interpretation appropriate?

Partly

Are all the source data underlying the results available to ensure full reproducibility?

Yes

Is the study design appropriate and is the work technically sound?

Partly

Are the conclusions drawn adequately supported by the results?

Partly

Are sufficient details of methods and analysis provided to allow replication by others?

Yes

Reviewer Expertise:

Interaction between viruses and hosts; mosquito-borne flavivirus; vaccine

I confirm that I have read this submission and believe that I have an appropriate level of expertise to confirm that it is of an acceptable scientific standard, however I have significant reservations, as outlined above.

# Response

**Published:** 2020-06-25

**Authors:** Pyae Phyo Kyaw

**Affiliations:** Department of Medical Research, Myanmar

1. The whole article was simple as a scientific research. More in-depth analysis of data is recommended. More comparison should be conducted to reflect the details in multi-aspects. For example, "parents/guardians had poor knowledge of JE" is sentenced. Since the details of the guardians were investigated, the correlation should be conducted, like with age? or occupations? or else? or none. Besides, are there any differences in awareness between urban and rural places?...

 Response : Thank you for your suggestion and we performed more in-depth analysis to determine the socio-demographic characteristics associated with good knowledge score and presented in the table-3. (line 218-234) We also performed an in-depth analysis to determine the good knowledge score with the child being vaccinated and presented in the table-4. (line 239-244)

 2. To fully reflect the effect of public awareness in Yangon, authors should not choose nine townships randomly. Especially the locations are concentrated. The choices need to be more meaningful, like the widest vector distribution ones, the most severely suffered ones. OR, at least, the details of these nine townships should be presented, like incidence cases.

 Response : We believe that random selection of township would provide generalizable information for the Yangon region. If we selected based on the incidence rate of JE cases or vector distribution, the knowledge, perception and vaccination coverage would not be representative to the population in Yangon. We hope this is fine. (line 78-90)

 3. The complex relationship between JEV and congeneric members, like DENV and ZIKV, is widely proposed. Since dengue is prevalent locally, the vaccination against Flavivirus members or infections should be also considered in the questionnaire.

 Response: In Myanmar, we have no vaccination programme against Dengue and Flavivirus infections and we did not have the opportunity to explore about this.

 4. In table 1, (1) is single parent family excluded? (2) what does "Do not know family members suffered JE" exactly mean? do not know the symptoms or do not know the children's conditions?

 Response:

 (1) Single parent family was not excluded, considered under nuclear family. In type of family, Nuclear family means family which has father or mother with their children. Extended family means family which has either father, mother or grandfather, grandmother or uncle, aunty with children. We added this in the footnote of the table. (line 159-161)

 (2) "Do not know family members suffered JE" means parents/guardians did not know their children have previous history of infection with JE virus. (We removed that variable from table-1 as the author group feels it does not add valuable information)

 5. "23% did not know the symptoms of JE" is sentenced. So, how will these parents know their kids are not suffered from JE? Is the "0" morbidity presented by parents credible?

 Response: The answer by the participant to the question on JE morbidity by asking whether the family member suffered JE before was based on the clinic or hospital record. So it is possible that the participant can report whether or not their children suffered JE even they did not know the symptoms of JE. Anyway, I removed that variable from table 1 as the author group feels it does not add valuable information.
